# Small-Molecule-Induced Activation of Cellular Respiration Inhibits Biofilm Formation and Triggers Metabolic Remodeling in Staphylococcus aureus

**DOI:** 10.1128/mbio.00845-22

**Published:** 2022-07-19

**Authors:** Ken-ichi Okuda, Satomi Yamada-Ueno, Yutaka Yoshii, Tetsuo Nagano, Takayoshi Okabe, Hirotatsu Kojima, Yoshimitsu Mizunoe, Yuki Kinjo

**Affiliations:** a Department of Bacteriology, The Jikei University School of Medicine, Minato-ku, Tokyo, Japan; b Jikei Center for Biofilm Science and Technology, Minato-ku, Tokyo, Japan; c Division of Respiratory Diseases, Department of Internal Medicine, The Jikei University School of Medicine, Minato-ku, Tokyo, Japan; d Drug Discovery Initiative, The University of Tokyo, Bunkyo-ku, Tokyo, Japan; University of British Columbia

**Keywords:** biofilms, *Staphylococcus aureus*, high-throughput screening, cellular respiration, aminoglycoside

## Abstract

Staphylococcus aureus, a major pathogen of community-acquired and nosocomial-associated infections, forms biofilms consisting of extracellular matrix-embedded cell aggregates. S. aureus biofilm formation on implanted medical devices can cause local and systemic infections due to the dispersion of cells from the biofilms. Usually, conventional antibiotic treatments are not effective against biofilm-related infections, and there is no effective treatment other than removing the contaminated devices. Therefore, the development of new therapeutic agents to combat biofilm-related infections is urgently needed. We conducted high-throughput screening of S. aureus biofilm inhibitors and obtained a small compound, JBD1. JBD1 strongly inhibits biofilm formation of S. aureus, including methicillin-resistant strains. In addition, JBD1 activated the respiratory activity of S. aureus cells and increased the sensitivity to aminoglycosides. Furthermore, it was shown that the metabolic profile of S. aureus was significantly altered in the presence of JBD1 and that metabolic remodeling was induced. Surprisingly, these JBD1-induced phenotypes were blocked by adding an excess amount of the electron carrier menaquinone to suppress respiratory activation. These results indicate that JBD1 induces biofilm inhibition and metabolic remodeling through respiratory activation. This study demonstrates that compounds that enhance the respiratory activity of S. aureus may be potential leads in the development of therapeutic agents for chronic S. aureus-biofilm-related infections.

## INTRODUCTION

Biofilms are clusters of microbial cells covered with an extracellular matrix (ECM) comprising biomolecules such as DNA, polysaccharides, and proteins (1–3). Dispersion of cells from the mature biofilm facilitates a new biofilm development cycle at new colonization sites (4, 5). Biofilms formed on surfaces of medical devices cause biofilm-related infections (6, 7). Bacterial cells in the biofilm show low metabolic activity; therefore, they are difficult to eradicate with antibiotics that are effective against dividing bacterial cells (8). At present, biofilm-related infections associated with implanted medical devices are usually treated by surgically removing the contaminated devices combined with antibiotic therapy (9). However, the removal of devices may present an increased risk of complications (10, 11). Therefore, a better method to control biofilm formation by pathogenic bacteria is a significant, unmet medical need.

Staphylococcus aureus, a major human pathogen (12), forms biofilms on medical devices, such as catheters, pacemakers, and prosthetic joints (6). S. aureus produces extracellular DNA (eDNA), proteins, and polysaccharide intercellular adhesins (PIAs) as ECM components, with the components important for biofilm formation varying among strains (13–17). Cell lysis (18–20), membrane vesicle-mediated export (17, 21, 22), and cell wall homeostasis (14, 23) are reportedly involved in DNA and/or cytoplasmic protein-dependent biofilm formation. PIA production is well studied and known to be governed by the *ica* operon (24, 25).

There have been several reports on large-scale screening of biofilm inhibitors. Opperman et al. (26) performed high-throughput screening (HTS) of small molecules that inhibited staphylococcal biofilm formation and obtained several aryl rhodanines that effectively inhibit staphylococcal and enterococcal biofilm formation. A novel benzimidazole molecule, known as ABC-1, was identified by HTS of small molecules inhibiting biofilm formation by Vibrio cholerae. ABC-1 was found to inhibit biofilm formation by multiple Gram-negative and Gram-positive bacterial pathogens, including Pseudomonas aeruginosa and S. aureus (27). Norgestimate, a synthetic progestin of the 19-norsteroid series, inhibits biofilm formation by S. aureus, including methicillin-resistant S. aureus (MRSA), by inhibiting the production of ECM components and resensitizing MRSA to β-lactam antibiotics (28).

Here, we aimed to obtain a new biofilm inhibitor against S. aureus by HTS using a cell-based phenotypic screening method and to understand the molecular mechanism underlying S. aureus biofilm formation through a comprehensive analysis of the compound’s mechanisms of action.

## RESULTS

### Identification of a novel staphylococcal biofilm inhibitor, JBD1.

**FIG 1:**
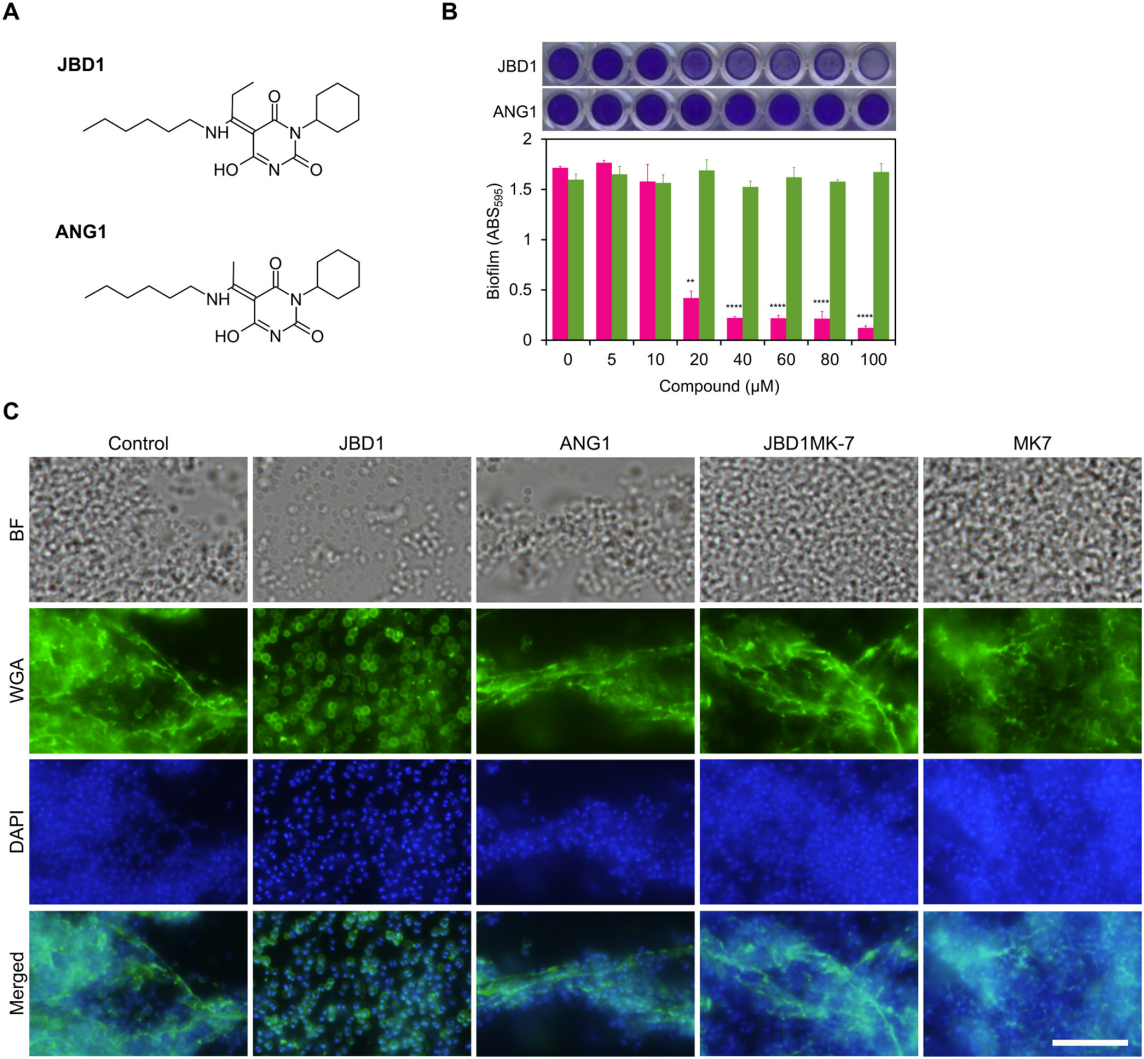
Biofilm-inhibitory activity of JBD1. (A) Structure of JBD1 and ANG1. (B) Biofilm-inhibitory activities of JBD1 (magenta) and ANG1 (green). The quantitative values of the crystal-violet-stained S. aureus SH1000 biofilms formed on 96-well plates are shown. Data represent the means with standard error from three independent experiments. Mean values were compared against 0 μM via one-way ANOVA. **, *P* < 0.01, ****, *P* < 0.0001 (Dunnett’s multiple-comparison test). Photographs of representative wells are shown above the graphs. (C) Imaging of fluorescently labeled PIA. S. aureus SH1000 was cultured in the presence of 5% DMSO (Control), 20 μM JBD1 (JBD1), 20 μM ANG1 (ANG1), 20 μM JBD1 and 100 μM MK-7 (JBD1MK-7), and 100 μM MK-7 (MK-7). PIA and DNA were stained using WGA-Alexa488 and DAPI, respectively. BF, bright field. Bar, 10 μm.

We performed HTS of biofilm inhibitors using a small molecule library consisting of 9,600 compounds. Compounds that inhibited biofilm formation but not cell growth were regarded as hit compounds because such compounds are expected to have a lower risk of inducing resistant strains. One of the hit compounds, (5E)-3-cyclohexyl-5-[1-(hexylamino)propylidene]-6-hydroxypyrimidine-2,4(3H,5H)-dione, named JBD1 (Fig. 1A), dose-dependently inhibited biofilm formation by S. aureus SH1000 (Fig. 1B). The CFU of the biofilms formed in the presence of JBD1 was significantly lower than that of control (Fig. S1A). JBD1 did not disrupt mature biofilms (Fig. S1B). Measurement of cell growth in the presence or absence of compounds confirmed that JBD1 does not inhibit cell growth (Fig. S2). JBD1 inhibited biofilm formation by all the staphylococcal strains tested, including four methicillin-sensitive S. aureus (MSSA) strains, four methicillin-resistant S. aureus (MRSA) strains, and two strains of S. epidermidis (Table 1) with IC_50_ values of 5.1 to 38.5 μM (Table 2). To clarify the structure-activity relationship of JBD1, 24 structural analogs of JBD1 were evaluated for biofilm-inhibitory activity (Fig. S3). Among the structural analogs, ANG2 and ANG20, which inhibited biofilm formation by SH1000 (IC_50_ = 54.8 and 39.0 μM, respectively), both possessed a propylidene group as well as JBD1. ANG1, a JBD1 analog that harbors an ethylidene group instead of the propylidene group, did not inhibit biofilm formation by SH1000 (Fig. 1B). ANG1 did not inhibit biofilm formation by all staphylococcal strains (IC_50_ > 100 μM), except for weakly inhibiting biofilm formation by S. aureus MR2 (IC_50_ = 56.6 μM) (Table 2). These results demonstrate that the propylidene group of JBD1 is critical for the biofilm-inhibitory activity.

**TABLE 1:** Strains and plasmids used in this study

Strain or plasmid	Description	Source or reference
Strains		
Staphylococcus aureus		
SH1000	S. aureus strain 8325-4 with functional *rsbU*	(48)
SH1000Δ*srrAB*	*srrA* and *srrB* were deleted from SH1000	This study
SH1000Δ*pflAB*	*pflA* and *pflB* were deleted from SH1000	This study
SH1000Δ*steT-ilvA1-ald1*	*steT*, *ilvA1*, and *ald1* were deleted from SH1000	This study
MS3	Clinical isolate of MSSA	(17)
MS9	Clinical isolate of MSSA	(17)
MS18	Clinical isolate of MSSA	(17)
MR2	Clinical isolate of MRSA	(17)
MR4	Clinical isolate of MRSA	(17)
MR10	Clinical isolate of MRSA	(17)
MR23	Clinical isolate of MRSA	(17)
USA300	Clinical isolate of MRSA	TCH1516
Staphylococcus epidermidis		
SE4	Clinical isolate of S. epidermidis	(17)
SE21	Clinical isolate of S. epidermidis	(17)
Escherichia coli		
DC10B	E. coli K12, Δ*dcm* mutant	(49)
Plasmids		
pIMAY	Plasmid for knockout of S. aureus genes by allelic exchange	(49)
pIMAYsrrAB	Upstream and downstream sequences of the *srrAB* cloned into pIMAY	This study
pIMAYpflAB	Upstream and downstream sequences of the *pflAB* cloned into pIMAY	This study
pIMAYsteT-ilvA1-ald1	Upstream and downstream sequences of the *steT-ilvA1-ald1* cloned into pIMAY	This study

**TABLE 2:** Biofilm inhibitory activities of JBD1 and ANG1 against staphylococcal strains

Strain	IC_50_^*a*^ value (μM)	
	JBD1	ANG1
MSSA		
SH1000	15.4	>100
MS3	38.5	>100
MS9	5.1	>100
MS18	14.6	>100
MRSA		
MR2	18.6	56.6
MR4	23.1	>100
MR10	7.7	>100
MR23	12.1	>100
Staphylococcus epidermidis		
SE4	15.8	>100
SE21	17.8	>100

a Half-maximal inhibitory concentration.

10.1128/mbio.00845-22.1 FIG S1 Effects of JBD1 on colony-forming units (CFUs) of biofilms (A) and stability of mature biofilms (B). (A) Biofilms of S. aureus SH1000 were formed in the presence and absence of JBD1 and MK-7, and the CFUs were measured. Mean values were compared via one-way ANOVA. *, *P* < 0.05 (Tukey’s multiple comparison test). (B) Mature biofilms of S. aureus SH1000 were treated with JBD1. The quantitative values of the crystal violet-stained biofilms on 96-well plates are shown. Data represent the means with standard error from three independent experiments. Mean values were compared via one-way ANOVA. **, *P* < 0.01, ****, *P* < 0.0001 (Tukey’s multiple comparison test). Download FIG S1, PDF file, 0.4 MB. Copyright © 2022 Okuda et al. 2022 Okuda et al. https://creativecommons.org/licenses/by/4.0/ This content is distributed under the terms of the Creative Commons Attribution 4.0 International license.

10.1128/mbio.00845-22.2 FIG S2 Effects of JBD1 and ANG1 on cell growth. (A) Absorbance of S. aureus SH1000 after culturing for 24 h in the presence or absence of JBD1 (magenta) or ANG1 (green). Data represent the means of three independent experiments with standard error. Mean values were compared against 0 μM via one-way ANOVA. (B) Growth curves of S. aureus SH1000 in the presence of 5% DMSO (blue), 20 μM JBD1 (magenta), 20 μM ANG1 (green), 20 μM JBD1 and 100 μM MK-7 (orange), and 100 μM MK-7 (gray). Data represent the means of three independent experiments with standard error (error bars are smaller than the size of the symbols). Download FIG S2, PDF file, 0.1 MB. Copyright © 2022 Okuda et al. 2022 Okuda et al. https://creativecommons.org/licenses/by/4.0/ This content is distributed under the terms of the Creative Commons Attribution 4.0 International license.

10.1128/mbio.00845-22.3 FIG S3 Structure-activity relationships of biofilm inhibitors. Chemical structures of JBD1 and 24 structural analogs. The values in parentheses indicate biofilm-inhibitory activity against S. aureus SH1000 (IC_50_, μM). Download FIG S3, PDF file, 1.5 MB. Copyright © 2022 Okuda et al. 2022 Okuda et al. https://creativecommons.org/licenses/by/4.0/ This content is distributed under the terms of the Creative Commons Attribution 4.0 International license.

SH1000 produces PIA in biofilms (13, 28, 29); therefore, the effects of JBD1 and ANG1 on PIA production were analyzed by fluorescence microscopy (Fig. 1C and Fig. S4). In the absence of the compounds, PIA stained with wheat germ agglutinin (WGA) Alexa Fluor 488 conjugate (WGA-Alexa488) was observed as fibrous structures connecting cells. The fibrous structures were also observed in the presence of ANG1 but not in the presence of JBD1. Previously, we confirmed that these fibrous structures disappear after treatment with dispersin B (28), a PIA-degrading β-*N*-acetylglucosaminidase (30). These results indicate that JBD1 inhibits synthesis of PIA.

10.1128/mbio.00845-22.4 FIG S4 Imaging of fluorescently labeled PIA. PIA and DNA of S. aureus SH1000 were stained with WGA-Alexa488 and DAPI, respectively. Each panel in Fig. 1C is an enlargement of the white frame. Bar, 20 μm. Download FIG S4, PDF file, 2.6 MB. Copyright © 2022 Okuda et al. 2022 Okuda et al. https://creativecommons.org/licenses/by/4.0/ This content is distributed under the terms of the Creative Commons Attribution 4.0 International license.

Next, we observed the three-dimensional structures of the biofilms formed in the presence or absence of JBD1 using confocal laser scanning microscopy (CLSM) after staining live and dead cells with fluorescence probes SYTO9 and PI, respectively (Fig. S5). Under control conditions, the biofilm exhibited a thickness of approximately 50 μm. Live cells accounted for most cells, and dead cells were localized throughout the biofilm as a minor population. In the presence of JBD1, the biofilm was thinner, with a small number of live cells attached to the bottom surface and no dead cells.

10.1128/mbio.00845-22.5 FIG S5 Confocal laser scanning microscopy (CLSM) images of biofilms. Biofilms of S. aureus SH1000 formed in the absence of a compound (the control) and the presence of 50 μM JBD1 (JBD1) or 50 μM JBD1 and 100 μM MK-7 (JBD1MK-7) were stained with SYTO9 (green) and PI (red), which stain live and dead cells, respectively. Three-dimensional structures of the biofilms were observed using CLSM. Download FIG S5, PDF file, 2.5 MB. Copyright © 2022 Okuda et al. 2022 Okuda et al. https://creativecommons.org/licenses/by/4.0/ This content is distributed under the terms of the Creative Commons Attribution 4.0 International license.

### JBD1 induces sensitization to aminoglycosides by activating the respiratory chain.

Bacteria in biofilms are resistant and/or tolerant to antibacterial agents by virtue of mechanisms such as reduced metabolic activity and reduced penetration to drugs imposed by the matrix (31, 32). Therefore, we investigated the influence of JBD1 on the antibiotic susceptibility of the staphylococcal strains under culture conditions that promote biofilm formation. Four-fold or greater differences in MIC values were regarded as significant (Table 3). In experiments using SH1000, JBD1 increased the susceptibility (a 4-fold decrease in MIC values) to the three aminoglycosides (gentamicin, kanamycin, and tobramycin) and one β-lactam (ampicillin), whereas ANG1 only increased the susceptibility to ampicillin. To confirm the generality of aminoglycoside-sensitization by JBD1 in S. aureus, experiments using the clinically isolated MSSA strain (MS9) and the MRSA strains (MR4, MR10, and USA300) were performed. JBD1, but not ANG, increased susceptibility to the aminoglycosides (a 4- to 8-fold decrease in MIC values) (Table S1). Taken together, we hypothesize that biofilm inhibition and aminoglycoside-sensitization of S. aureus are induced by JBD1 through a common mechanism.

**TABLE 3:** MICs of antibiotics against S. aureus SH1000 in the presence or absence of biofilm inhibitors^*a*^

Antibiotic	Control	JBD1	ANG1	JBD1MK-7	MK-7
Gentamicin	16	4	16	16	16
Kanamycin	64	16	64	64	64
Neomycin	64	32	64	64	64
Tobramycin	16	4	16	16	16
Ampicillin	1	0.25	0.25	N.D.^*b*^	N.D.
Oxacillin	1	0.5	0.5	N.D.	N.D.
Cefazolin	1	0.5	1	N.D.	N.D.
Cefmetazole	2	2	2	N.D.	N.D.
Cefotaxime	4	2	2	N.D.	N.D.
Erythromycin	0.25	0.125	0.25	N.D.	N.D.
Tetracycline	0.5	0.5	0.5	N.D.	N.D.
Fosfomycin	32	32	16	N.D.	N.D.
Vancomycin	8	4	4	N.D.	N.D.

a MICs are in μg/mL.

b N.D., not determined.

10.1128/mbio.00845-22.7 TABLE S1 MICs (μg/mL) of antibiotics against S. aureus strains in the presence or absence of compounds. Download Table S1, PDF file, 0.10 MB. Copyright © 2022 Okuda et al. 2022 Okuda et al. https://creativecommons.org/licenses/by/4.0/ This content is distributed under the terms of the Creative Commons Attribution 4.0 International license.

**FIG 2:**
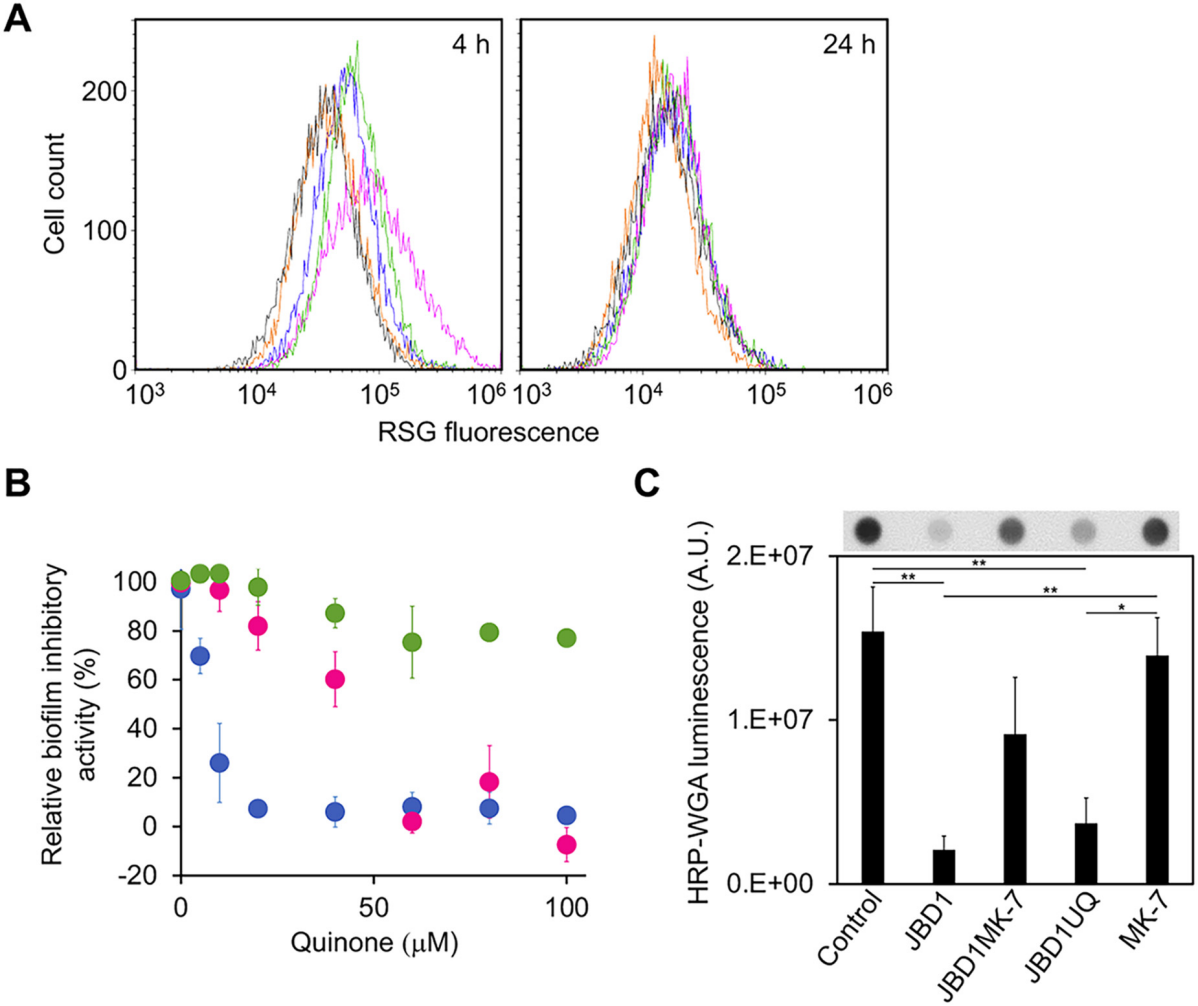
Relationship between respiratory activation and biofilm-inhibitory effects of JBD1. (A) Respiratory activity of S. aureus SH1000. Cells cultured for 4 h or 24 h in the absence of compound (blue), presence of 20 μM JBD1 (magenta), 20 μM ANG1 (green), 20 μM JBD1 and 100 μM MK-7 (orange), or 100 μM MK-7 (gray) were stained with RSG and analyzed using a flow cytometer. (B) Effects of quinones on biofilm-inhibitory activities of JBD1 against S. aureus SH1000. The inhibitory activity of 20 μM JBD1 in the absence of quinone was set to 100%, and the relative values in the presence of MK-7 (blue), MK9 (magenta), or UQ (green) were plotted. Data are the means with standard error from three independent experiments. (C) Effects of JBD1 and quinones on PIA production by S. aureus SH1000. Cells were cultured in the presence of 5% DMSO (Control), 20 μM JBD1 (JBD1), 20 μM JBD1 and 100 μM MK-7 (JBD1MK-7), 20 μM JBD1 and 100 μM UQ (JBD1UQ), and 100 μM MK-7 (MK-7). PIA was quantified by dot blot analysis using WGA-horseradish peroxidase. Bar graphs show the quantitative values of the signal intensity. Data represent means with standard error from three independent experiments. Mean values were compared via one-way ANOVA. *, *P* < 0.05, **, *P* < 0.01 (Tukey's multiple comparison test). Photographs of representative signal images are shown above the graphs.

Bacterial uptake of aminoglycosides is dependent on a proton motive force (PMF) acting across the cell membrane (33). The PMF is generated in response to the transport of protons outside the membrane via the action of the respiratory chain. Therefore, we hypothesized that activation of the respiratory chain by JBD1 improves aminoglycoside sensitivity. The respiratory activity of cells cultured in the presence/absence of JBD1 or ANG1 (20 μM each) was measured using RedoxSensor green (RSG), a fluorescent probe whose intensity depends on bacterial respiratory activity (34). The fluorescence intensity of the cells was measured using a flow cytometer. In the presence of JBD1, the RSG-fluorescence intensity of 4-h cultured cells was remarkably stronger than that of control cells (Fig. 2A), suggesting that JBD1 enhanced respiratory activity. Furthermore, the fluorescence intensity of the ANG1-treated cells was markedly lower than that of JBD1-treated cells and only slightly stronger than that of the control cells. Under our experimental conditions, at 4 h of growth, control cells formed biofilms (approximately 50% of the 24-h biofilm). JBD1, but not ANG1, showed a biofilm-inhibitory effect at 4 h of growth (Fig. S6). There was no difference in respiratory activity among the 24-h cultured cell samples (Fig. 2A), suggesting that JBD1 exerts its effects in the early stages of growth.

10.1128/mbio.00845-22.6 FIG S6 Effects of compounds on biofilm formation at 4 h of growth. S. aureus SH1000 biofilms were formed for 4 h in the presence of 5% DMSO (the control), 20 μM JBD1 (JBD1), 20 μM ANG1 (ANG1), 20 μM JBD1 and 100 μM MK-7 (JBD1MK-7), and 100 μM MK-7 (MK-7). The quantitative values of the crystal violet-stained S. aureus SH1000 biofilms formed on 96-well plates are shown. Mean values were compared via one-way ANOVA. *, *P* < 0.05, ****, *P* < 0.0001 (Tukey’s multiple comparison test). Download FIG S6, PDF file, 0.1 MB. Copyright © 2022 Okuda et al. 2022 Okuda et al. https://creativecommons.org/licenses/by/4.0/ This content is distributed under the terms of the Creative Commons Attribution 4.0 International license.

The electron carrier in Gram-positive bacteria, including S. aureus, is a menaquinone (MK), a common electron carrier in the respiratory chains of Gram-positive bacteria (35). MK, which is reduced via NADH dehydrogenase (NDH) action, transfers electrons to the respiratory chain (36). We hypothesized that the exogenous addition of an excessive amount of MK alters the electron transport chain balance, thereby affecting the JBD1-mediated activation of respiration. S. aureus produces MKs with isoprenoid side chains of 7 to 10 (MK-X; where X is the number of side chains) (37). We investigated the effect of an excessive amount of MK-7 (100 μM) on the ability of JBD1 to enhance the respiratory activity of SH1000. In 4-h-cultured cells, the addition of MK-7 alone slightly suppressed the respiratory activity compared to the control. In the presence of both JBD1 and MK-7 (JBD1MK-7), the effect of JBD1 with respect to activating respiration was lost (Fig. 2A). MK-7 addition did not affect cell growth of SH1000 during the log phase (Fig. S2B). These results indicate that an excessive amount of MK inhibits the promotion of respiratory activity induced by JBD1. We next investigated the effect of MK-7 on aminoglycoside sensitization by JBD1. There was no significant difference in aminoglycoside susceptibility between the control and JBD1MK-7-treated strains. MK-7 alone did not significantly alter the aminoglycoside susceptibility of all strains (Table 3 and Table S1). These results demonstrate that JBD1 increases PMF by promoting cellular respiration, thereby enhancing the aminoglycoside uptake, leading to sensitization.

### Respiratory chain activation is required for JBD1 to inhibit biofilm formation.

Next, we investigated whether inhibition of JBD1-dependent respiratory activation by MK-7 affects the biofilm-inhibitory activity of JBD1. We assayed the ability of JBD1 to inhibit biofilm formation at 24 h of growth in the presence of MK-7 at different concentrations (Fig. 2B). MK-7 suppressed the biofilm-inhibitory activity of JBD1 in a dose-dependent manner. We additionally investigated the effects of different quinones. On comparing MK-7 and MK-9, which have different lengths of side chains, MK-7 was more effective at suppressing the biofilm-inhibitory activity of JBD1. Ubiquinone (UQ), an electron carrier used by most Gram-negative bacteria but not by S. aureus (35, 37), did not affect the ability of JBD1 to inhibit biofilm formation. The biofilm-inhibitory effect of JBD1 at 4 h of growth was also counteracted by the addition of MK-7 (Fig. S6). These results indicate that the inhibitory activity of JBD1 on biofilm formation is effectively suppressed by the quinone typically used in S. aureus. As revealed by CLSM, a biofilm with almost the same thickness as the control was formed in the presence of JBD1MK-7, and the distribution of a small number of dead cells throughout the biofilm was also confirmed (Fig. S5). Next, the effects of MK-7 on PIA production were analyzed by fluorescence microscopy. In the presence of JBD1MK-7, the formation of PIA fibrous structures was observed to be similar to that observed in the control conditions (Fig. 1C and Fig. S4), indicating that MK-7 counteracts the inhibitory effect of JBD1 on PIA production. When extracellular PIA was quantified using the dot blot analysis, the amount of PIA produced was significantly decreased in JBD1 and JBD1UQ compared with the control. However, no significant difference was observed between the control and JBD1MK7 (Fig. 2C). Taken together, the structural and biochemical properties of the biofilm formed in the presence of JBD1MK-7 were similar to those of the control. Considering the difference in respiratory activity under each condition as shown above (Fig. 2A), it was concluded that the promotion of respiratory activity by JBD1 triggers the inhibition of biofilm formation.

### Effect of JBD1 on the gene expression profile.

**FIG 3:**
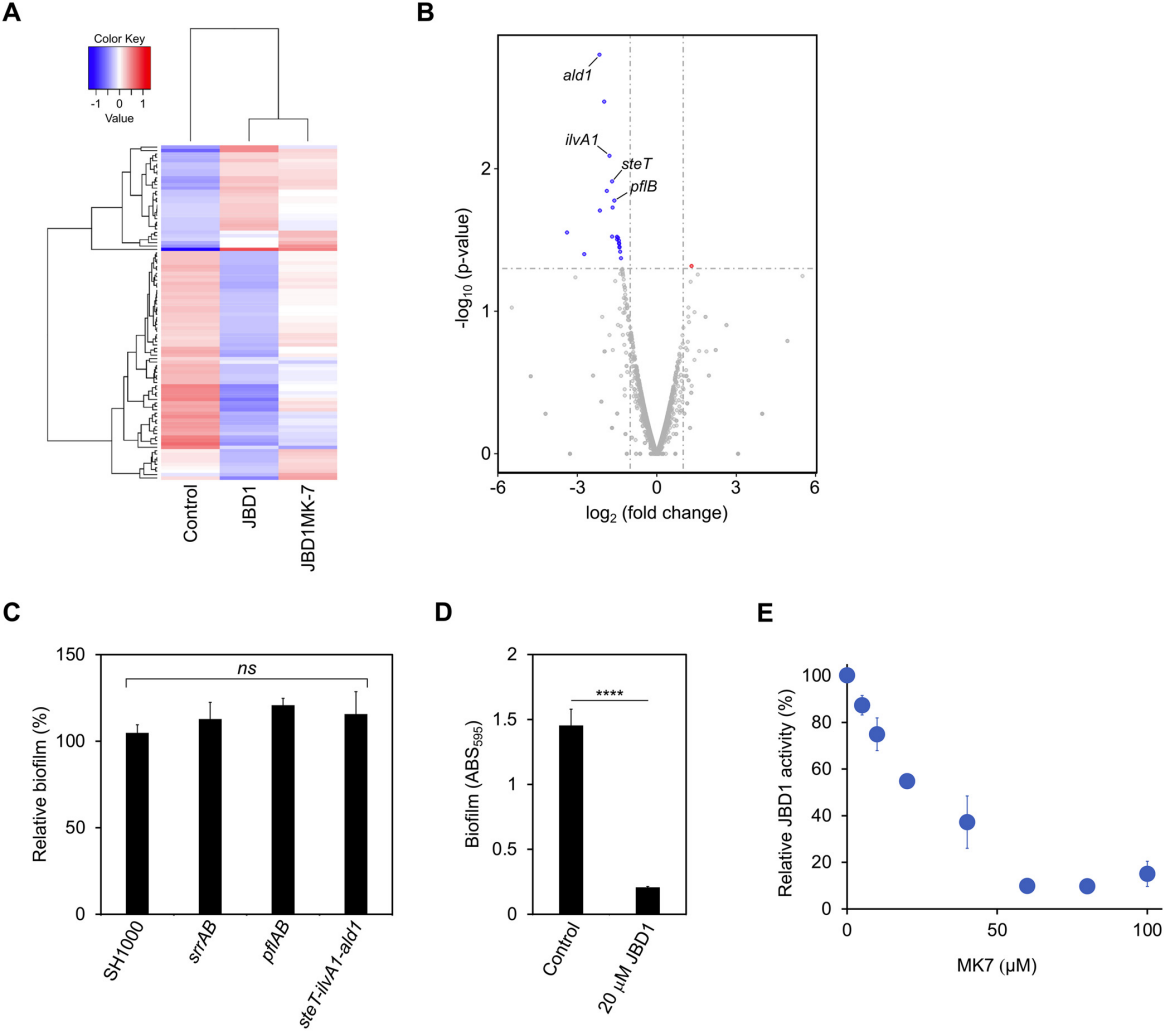
Effect of JBD1 on the gene expression profile. (A) Cluster analysis of differentially expressed genes. Total RNA was extracted from S. aureus SH1000 cultured in the presence/absence of JBD1 or JBD1MK-7 and analyzed by RNA-sequencing. Genes with high expression are shown in red, and those with low expression in blue. (B) Differential expression volcano plot. Red and blue dots represent genes that are significantly upregulated and downregulated, respectively. *x* axis: log_2_ fold change of gene expression. *y* axis: statistical significance of the differential expression in log_10_ (*P*-value). Among the 22 genes whose expression was significantly changed (*P* < 0.05), a red dot indicates those with increased expression (log_2_ > 1), while those with decreased expression (log_2_ < −1) are indicated by blue dots. (C) Biofilms of S. aureus SH1000 and mutant derivatives (*srrAB*, *pflAB*, *steT*-*ilvA1*-*ald1*) were formed on 96-well plates. The *y* axis represents the relative values with respect to the quantitative value of the crystal violet-stained WT biofilm, designated as 100%. Data represent means with standard error from three independent experiments. Mean values were compared via one-way ANOVA. (D) Quantitative values of the crystal-violet-stained biofilms of the *srrAB* mutant formed on 96-well plates in the presence or absence of 20 μM JBD1. Data represent means with standard error from three independent experiments, ****, *P* < 0.0001 (*unpaired t test*). (E) Effects of MK-7 on the biofilm-inhibitory activities of JBD1 against the *srrAB* mutant. The inhibitory activity of 20 μM JBD1 in the absence of quinone was set to 100%, and the relative values in the presence of MK-7 were plotted. Data represent means with standard error from three independent experiments.

As JBD1 induced multiple phenotypic changes, it was hypothesized that it would also affect the gene expression profile of S. aureus. To investigate this, we sequenced total RNA extracted from S. aureus cells cultured in the presence/absence of JBD1 or JBD1MK-7 for 4 h during the early log phase. The overall expression profiles of the control, JBD1-treated, and JBD1MK-7-treated cells are displayed as a heat map (Fig. 3A). A large difference was observed in the gene expression profiles between the control and JBD1, but the difference was limited when comparing JBD1 and JBD1MK-7. As the multiple phenotypes induced by JBD1 are suppressed in the presence of MK-7, we hypothesized that genes expressed at different levels between JBD1 and JBD1MK-7 are involved in determining the observed phenotype of JBD1-treated cells. We identified 21 genes that were significantly downregulated in JBD1-treated cells (compared to JBD1MK7-treated cells) (Fig. 3B and Table S2). We focused on the fact that 4 of the 10 genes, excluding the 11 genes that encode hypothetical proteins from the 21 genes, encode metabolic proteins. These included genes involved in amino acid metabolism; *steT* (SAOUHSC_01450) encoding an amino acid permease, *ilvA1* (SAOUHSC_01451) encoding a threonine dehydratase, and *ald1* (SAOUHSC_01452) encoding an alanine dehydrogenase, suggesting that JBD1 affects the amino acid profile of S. aureus. Expression of *pflB* (SAOUHSC_00187), which encodes an enzyme that converts pyruvate to formic acid and acetyl-CoA, was also decreased in JBD1-treated cells compared to that in JBD1MK-7-treated cells (Table 4). It has been reported that *pfl* expression is enhanced in biofilms (compared to planktonic cells) (29), and our results are in good agreement with this report. Expression levels of the above genes were also estimated by real-time PCR (Table 4). The expression of *pflA* (SAOUHSC_00188), which encodes a PflB-activating enzyme, was also determined using real-time PCR because *pflA* is located in the same operon as *pflB*. Real-time PCR using total RNA extracted from the cells after 4 h of culture (early log phase; Fig. S2B) revealed that the expression levels of each gene in JBD1-treated cells were significantly lower than those in JBD1MK-7-treated cells (Table 4). Furthermore, in real-time PCR using total RNA extracted from the cells after 8 h of culture (log phase; Fig. S2B), similar results were obtained (Table 4). These results confirm the reliability of the results of the RNA-sequencing analysis and show that differences in the expression profiles of metabolic genes are maintained at least until, and throughout, the log phase of growth.

**TABLE 4:** Expression levels of metabolic genes in JBD1- and JBD1MK-7-treated cells

Locus tag	Gene	Product	Fold change (JBD1/JBD1MK-7)		
			RNA-seq	Real-time PCR (4 h)	Real-time PCR (8 h)
SAOUHSC_01450	*steT*	Amino acid permease	−3.2	−3.1 ± 0.4	−2.6 ± 0.4
SAOUHSC_01451	*ilvA1*	Threonine dehydratase	−3.4	−2.8 ± 0.7	−2.7 ± 0.4
SAOUHSC_01452	*ald1*	Alanine dehydrogenase	−4.5	−3.5 ± 0.6	−3.0 ± 0.6
SAOUHSC_00187	*pflB*	Formate acetyltransferase	−3.0	−3.1 ± 0.3	−2.9 ± 0.3
SAOUHSC_00188	*pflA*	Pyruvate-formate-lyase activating enzyme	−2.6	−2.2 ± 0.2	−2.6 ± 0.4

10.1128/mbio.00845-22.8 TABLE S2 RNA sequencing data related to Fig. 3. Download Table S2, XLSX file, 0.01 MB. Copyright © 2022 Okuda et al. 2022 Okuda et al. https://creativecommons.org/licenses/by/4.0/ This content is distributed under the terms of the Creative Commons Attribution 4.0 International license.

### Effects of gene knockout on biofilm formation.

The reduced expression of specific genes and the inhibition of biofilm formation caused by JBD1 are results consistent with the reported phenotypes of *srrAB*-deficient strains (38–41). Therefore, we constructed a *srrAB*-deficient strain and compared the degree of biofilm formation with that of the wild-type strain. No significant difference was observed in the degree of biofilm formation under our experimental conditions (Fig. 3C). JBD1 exhibited an inhibitory activity with respect to biofilm formation in the *srrAB*-deficient strain as well as in the wild type (Fig. 3D). Furthermore, the inhibitory activity of JBD1 against biofilm formation in the *srrAB*-deficient strain was inhibited by MK-7 in a concentration-dependent manner (Fig. 3E). These results revealed that the *srrAB* system is not essential for JBD1 to exert its activity. To investigate the roles played by metabolism-related genes in S. aureus biofilm formation, we constructed deletion mutants of *steT*-*ilvA1*-*ald1* and *pflAB*. No significant difference was observed in the amount of biofilm between the WT and the mutants (Fig. 3C). This suggests that the inhibition of biofilm formation by JBD1 cannot solely be explained by the decreased expression of specific metabolism-related genes.

### JBD1 induces metabolic remodeling through respiratory chain activation.

**FIG 4:**
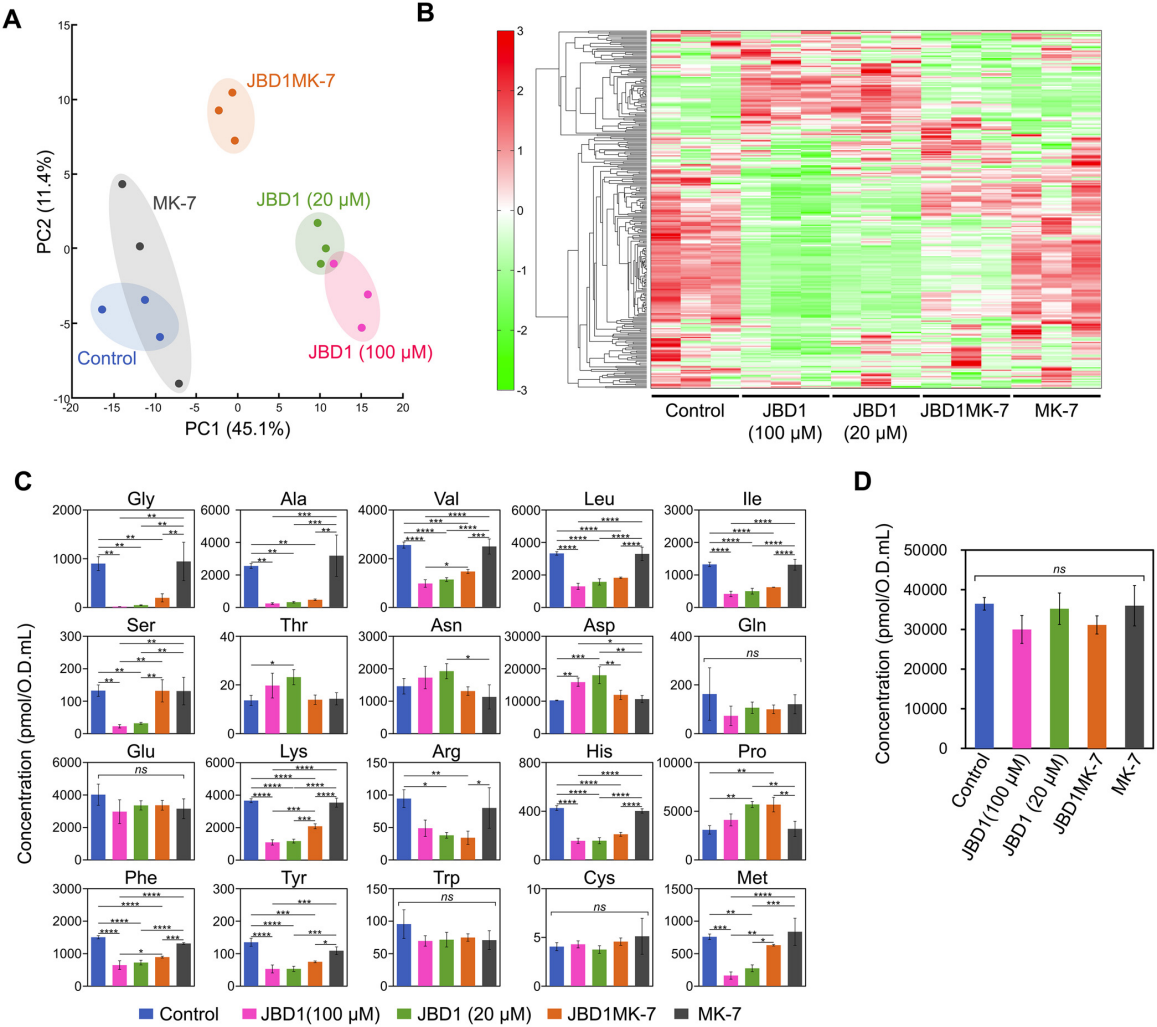
Effect of JBD1 on metabolic profile. (A) PCA score plot of metabolites in S. aureus SH1000 cultured in the presence or absence of compounds. Control, 5% DMSO; JBD1 (100 μM), 100 μM JBD1; JBD1 (20 μM), 20 μM JBD1; JBD1MK-7, 20 μM JBD1 and 100 μM MK-7; MK-7; 100 μM MK-7. (B) Heat map representation of the metabolome profile analyzed by hierarchical clustering analysis. The distances between peaks are displayed in tree diagrams. Red and green colors indicate higher and lower contents of the metabolites, respectively. (C) Quantitative values of each amino acid. *y* axis represents the amount of the amino acid (pmol) in cells from 1 mL of the cell suspension of OD_595_ = 1. Data represent means with standard error from three independent experiments. Mean values were compared via one-way ANOVA. *, *P* < 0.05, **, *P* < 0.01, ***, *P* < 0.001, ****, *P* < 0.0001 (Tukey’s multiple comparison test). (D) Quantitative values of total amino acids. *y* axis represents the amount of total amino acids (pmol) in cells from 1 mL of the cell suspension of OD_595_ = 1. Data represent means with standard error from three independent experiments. Mean values were compared via one-way ANOVA.

After noting the altered expression of multiple metabolism-related genes in the presence of JBD1, we hypothesized that JBD1 influences the metabolic profile of S. aureus. To investigate this, we extracted metabolites from cells cultured in the presence/absence of JBD1 or JBD1MK-7 and analyzed the metabolomic profiles. Detailed metabolomic data are shown in Table S3. Principal-component analysis (PCA) of the metabolomic data revealed clear clustering under different treatment conditions (Fig. 4A). The first and second principal components (PC1 and PC2) accounted for 45.1% and 11.4% of the total variance, respectively. Differences in the amount of biofilm formed under each treatment condition showed a clear correlation with PC1. Control and MK7-treated groups, which form substantial biofilms, were negatively aligned with PC1. Furthermore, the JBD1 (20 μM)- and JBD1 (100 μM)-treated groups, in which biofilm formation was inhibited, were positively aligned with PC1. Notably, the JBD1MK-7-treated group was aligned almost in the middle. Among the loading factors against PC1, nucleic acid-related substances and amino acid-related substances were abundant in metabolites with positive and negative contribution rates, respectively (Table S3). In the heat map representation of the hierarchical clustering analysis of the metabolomic data, the profiles of the control and MK-7-treated groups were markedly different from those of the JBD-1-treated groups, and JBD1MK-7 showed an intermediate metabolic profile (Fig. 4B). We noted that metabolites that differ between JBD and JBD1MK-7 included several amino acids (Table S3), and thus we compared the quantitative values of 20 different amino acids in each group (Fig. 4C). The concentration of threonine was significantly higher in the JBD1 (20 μM)-treatment group than that in the control and tended to be lower in the JBD1MK-7-treatment group than that in the JBD1 (20 μM)-treatment group (Fig. 4C). Similarly, the concentration of aspartate was significantly higher in the JBD1 (20 μM)-treatment group than that in the control and lower in the JBD1MK-7-treatment group than that in the JBD1 (20 μM)-treatment group (Fig. 4C). S. aureus expresses an enzyme that catabolizes the conversion of aspartate to oxaloacetate, a key intermediate in the TCA cycle (42). As NADH molecules generated by the TCA cycle areoxidized in the respiratory chain, increased aspartate levels may be related to increased respiratory activity in the presence of JBD1. Additionally, serine, lysine, and methionine amounts were lower in the JBD1-treatment group than in the control and higher in the JBD1MK-7-treated group than in the JBD1-treated group (Fig. 4C). The total amount of amino acids was calculated based on the quantitative value of each amino acid (Fig. 4D). With respect to individual amino acids, many amino acids were decreased in the presence of JBD1; however, no significant difference was observed in the total quantity of amino acids between groups, which can be attributed to the significant increase in aspartic acid concentration in the presence of JBD1. Aspartate was the most abundant amino acid; in the control and JBD1MK-7-treated cells, it accounts for approximately one-third of the total amino acids, and in the JBD1-treated cells, it accounts for more than one-half of the total amino acids. Taken together, JBD1 effectively induces metabolic remodeling of S. aureus, including changes in the amino acid balance of S. aureus. The effect was significantly suppressed in the presence of MK-7, suggesting that it is triggered upon the activation of respiration.

10.1128/mbio.00845-22.9 TABLE S3 Complete metabolomic data related to Fig. 4. Download Table S3, XLSX file, 0.5 MB. Copyright © 2022 Okuda et al. 2022 Okuda et al. https://creativecommons.org/licenses/by/4.0/ This content is distributed under the terms of the Creative Commons Attribution 4.0 International license.

## DISCUSSION

The novel biofilm inhibitor JBD1 differs in structure from any other staphylococcal biofilm inhibitor reported so far (26–28, 43–45). Concerning the biofilm-inhibitory activity, the importance of the methyl group in JBD1, as revealed by studying the relationship between structure and activity, is a useful starting point for synthetic development aimed at obtaining a compound with higher activity. JBD1 was obtained by phenotypic screening for inhibitory activity against biofilm formation by S. aureus. In the absence of information on target molecules, we comprehensively evaluated the effects of JBD1 on antimicrobial susceptibility, gene expression, and metabolic profile to elucidate the mechanism of action of these molecules. We found that JBD1 can enhance respiratory activity and that the addition of the electron carrier menaquinone to the medium counteracts the effects of JBD1 with respect to promoting respiratory activity and inhibiting biofilm formation. In order to determine whether such a mechanism of action is unique to JBD1 or common to other biofilm inhibitors, we investigated the effect of menaquinone on the biofilm-inhibitory activity of norgestimate, which we had previously identified via phenotype screening (28). We found that menaquinone did not affect the inhibitory activity of norgestimate with respect to biofilm formation (data not shown), suggesting that the mechanism of action of JBD1 and norgestimate differ. The enhancement of respiratory activity by JBD1 results in the sensitization of S. aureus toward aminoglycosides; no other instances of this behavior have been reported for previously described biofilm inhibitors, including norgestimate. Recently, it has been reported that rhamnolipid produced by Pseudomonas aeruginosa improves the sensitivity of S. aureus to aminoglycosides (46). However, rhamnolipid reportedly promotes the uptake of aminoglycosides independently of the PMF by affecting the surface charge, membrane fluidity, and permeability to low molecular weight compounds. In contrast, JBD1 acts via enhancing respiratory activity. Therefore, the mechanisms of action of JBD1 and rhamnolipid with respect to sensitization to aminoglycosides seem to be different.

Several studies have reported the relationship between respiratory activity and biofilm formation. *srrAB*, which is a two-component control system, senses the hypoxic state of cells based on the level of reduced menaquinone and controls the expression of metabolism-related genes important for survival and development in a hypoxic environment (38). It has been shown that *pflAB* expression is reduced in *srrAB*-deficient strains (38). Constitutive activation of SrrAB in response to mutations in *srrA* or *srrB* results in the upregulation of genes involved in pyruvate fermentation (*pflA* and *pflB*), amino acid fermentation (*ilvA1* and *ald1*), and amino acid transport (*steT*) (39). Additionally, a deletion mutant of *srrAB* had a reduced capability for biofilm formation (38, 40). Similarly, mutant screening by another research group revealed that *srrA* is important for PIA-independent biofilm formation (41). These findings regarding the phenotypes controlled by *srrAB* are in good agreement with the reduced expression of metabolism-related genes and the decreased biofilm formation observed in the presence of JBD1. Therefore, we hypothesized that JBD1 acts on *srrAB*. However, the loss of *srrAB* did not affect the biofilm formation under our experimental conditions or the biofilm-inhibitory activity of JBD1. Deletions of genes related to amino acid metabolism (*steT*, *ilvA1*, *ald1*), whose expression was downregulated by JBD1, also had no effect on biofilm formation. S. aureus not only synthesizes amino acids but also transports amino acids into the cell from the external environment (47). This may be why the deletion of specific genes involved in amino acid metabolism did not affect biofilm formation. Although the target molecules of JBD1 are unclear, we speculate that JBD1 may regulate the expression of these metabolism-related genes by acting on regulatory system(s).

JBD1 induced a large variation in the metabolic profile of S. aureus, and MK-7 significantly suppressed the effect. This result indicates that respiratory stimulation by JBD1 triggers metabolic remodeling as well as inhibition of biofilm formation. In addition, the PC1 in PCA correlates well with the extent of biofilm formed in each group, suggesting that biofilm control becomes possible by controlling the metabolic profile.

In summary, the compound JBD1, obtained by HTS, inhibited biofilm formation and induced metabolic remodeling by promoting increased respiratory activity of S. aureus. By clarifying the target molecule and mechanism of action of JBD1, it may be possible to develop a new method for controlling intractable biofilm-related infections.

## MATERIALS AND METHODS

### Bacterial strains and culture conditions.

Bacterial strains and plasmids used in this study are listed in Table 1. S. aureus and S. epidermidis strains were grown overnight in Brain Heart Infusion (BHI) broth (Becton, Dickinson, Franklin Lakes, NJ, USA) unless otherwise mentioned. All mutants were constructed from S. aureus SH1000 (48) by allelic exchange using a pIMAY plasmid (49). Briefly, approximately 500 bp upstream and downstream sequences of the relevant gene were amplified from S. aureus SH1000 genomic DNA by PCR using the primers listed in Table S4. The resulting PCR fragments were connected by overlap extension PCR. The linear fragments of pIMAY were amplified by inverse PCR using primers presented in Table S4. The two fragments were fused using an In-Fusion HD Cloning Kit (TaKaRa Bio, Otsu, Japan) according to the manufacturer’s protocol. The resulting plasmids were amplified in E. coli DC10B (49) and then introduced into S. aureus by electroporation. After the allelic exchange, the mutant strains generated were verified by sequencing to confirm that the entire open reading frame was accurately deleted.

10.1128/mbio.00845-22.10 TABLE S4 Primers used in this study. Download Table S4, XLSX file, 0.01 MB. Copyright © 2022 Okuda et al. 2022 Okuda et al. https://creativecommons.org/licenses/by/4.0/ This content is distributed under the terms of the Creative Commons Attribution 4.0 International license.

### Biofilm formation.

Overnight cultures of S. aureus and S. epidermidis grown in BHI were diluted 500-fold in BHI supplemented with 1% glucose (BHIG) broth, 5% DMSO, with or without test compounds. Aliquots of the cell suspensions (200 μL) were transferred to the wells of a 96-well microtiter plate (Corning Inc, Corning, NY, USA) and incubated for 4 to 24 h at 37°C under static conditions. Bacterial growth was assessed by measuring the absorbance of cultures at 595 nm using a microplate reader (Infinite 200 PRO; Tecan, Mannedorf, Switzerland). After removing culture supernatants and planktonic cells, biofilms that formed at the bottom of the wells were washed twice with 200 μL phosphate-buffered saline (PBS), to remove loosely adherent cells, dried, stained with 100 μL of 0.05% crystal violet for 1 min, and subsequently washed twice with PBS. Finally, biofilm formation was quantified by measuring the absorbance at 595 nm using a microplate reader (Infinite 200 PRO). As the biofilms can form at the bottom of the wells in a patchy manner, multiple reads (4 × 4) per well, using the Infinite 200 Pro, were used to obtain the average value in each well. The IC_50_ was calculated using the following formula: IC_50_ = 10^[log (A/B) × (50 − C)/(D − C) + log (B)]^, where A corresponds to concentrations of test compound directly above 50% inhibition; B corresponds concentrations of test compound directly below 50% inhibition; C is percent inhibition directly below 50% inhibition; and D is percent inhibition directly above 50% inhibition.

### High-throughput screening.

To identify compounds that inhibit biofilm formation in S. aureus SH1000, we conducted HTS of 9,600 compounds stocked at the Drug Discovery Initiative of The University of Tokyo. Biofilm formation and quantification under the conditions detailed above were conducted automatically using a custom-built FreedomEVO System (Tecan). The final concentration of each compound for screening was 20 μM. Compounds that inhibited bacterial growth by 20% or more were excluded from the hits.

### Biofilm eradication assay.

An overnight culture of S. aureus SH1000 was diluted 500-fold into BHIG broth, and 200-μL aliquots were cultured in 96-well flat-bottomed polystyrene plates (Corning) at 37°C for 24 h under static conditions. Then, JBD1 was added to the wells at a final concentration of 20 or 100 μM (5% final concentration of DMSO). After incubation at 37°C for 2 h, the biofilms were quantified as described above.

### CFU measurement of biofilms.

An overnight culture of S. aureus SH1000 was diluted 500-fold into BHIG broth containing 5% DMSO, with or without test compounds, and 2-mL aliquots were cultured in a 40-mm dish at 37°C for 24 h under static conditions. After removing the supernatants, the biofilms were washed twice with 2 mL PBS. The CFU was determined by plating each dilution on BHI agar.

### Bacterial growth curve.

An overnight culture of S. aureus SH1000 was diluted 500-fold into fresh BHIG broth 5% DMSO, with or without test compounds, and 200-μL aliquots were cultured in 96-well flat -bottomed polystyrene plates (Corning) at 37°C for 24 h under static conditions. The absorbance of each culture at 600 nm was measured every 30 min for 24 h using a Bio Microplate Reader HiTS (Scinics Corp., Tokyo, Japan).

### Fluorescent labeling of PIA.

For fluorescent imaging of extracellular PIA, S. aureus cells were stained using WGA-Alexa 488 (Invitrogen, Carlsbad, CA, USA) according to a previously published method (28), with modifications. An overnight culture of S. aureus SH1000 was diluted 500-fold in BHIG broth containing 5% DMSO, with or without test compounds (20 μM JBD1, 20 μM ANG1, 20 μM JBD1 and 100 μM MK-7, or 100 μM MK-7), and 2-mL aliquots were cultured in 12-well flat-bottomed polystyrene plates (Corning) at 37°C for 24 h under static conditions. After removing the supernatants, the sedimented cells were resuspended in PBS, spotted on glass slides, and left to dry at room temperature. Then, the cells were stained with 5 μg/mL WGA-Alexa 488 (Invitrogen) in PBS for 20 min at 37°C, washed twice with PBS, and subsequently stained with DAPI (Wako, Osaka, Japan) for 5 min at room temperature. Stained cells were mounted with the Vectashield mounting medium (Vector Laboratories, Burlingame, CA, USA) and observed using a fluorescence microscope (ECLIPSE E600; Nikon, Tokyo, Japan).

### CLSM imaging.

S. aureus SH1000 were cultured statically in 2 mL of BHIG broth containing 5% DMSO with or without JBD1 (50 μM) or JBD1 (50 μM) and MK-7 (100 μM) in a 35-mm glass-bottom dish (Matsunami, Osaka, Japan) at 37°C for 24 h. After removing the supernatants, the biofilms formed on the glass were washed three times with 300 μL of PBS and stained with 200 μL of Filmtracer LIVE/DEAD Biofilm Viability Kit (Invitrogen) according to the manufacturer’s protocol. The stained biofilm was washed twice with 200 μL of double-distilled water (DDW), mounted with ProLong Glass Antifade Mountant (Invitrogen), and observed using an LSM880 microscope (Carl Zeiss, Jena, Germany).

### Antibiotic susceptibility testing.

Overnight cultures of S. aureus strains were diluted 500-fold in BHIG broth containing 5% DMSO, with or without test compounds (20 μM JBD1, 20 μM ANG1, 20 μM JBD1 and 100 μM MK-7, or 100 μM MK-7). Aliquots of the cell suspensions (195 μL) were transferred to the wells of a 96-well microtiter plate (Corning) containing antimicrobial agents of appropriate concentrations (5 μL) and incubated for 24 h at 37°C under static conditions. Bacterial growth was assessed by measuring the optical density at 595 nm (OD_595_) using a microplate reader (Infinite 200 PRO). The minimum concentration of the antibiotic that suppresses the increase of OD_595_ to less than 20% from the start of culture was defined as the MIC.

### Measurement of respiratory activity.

An overnight culture of S. aureus SH1000 was diluted 500-fold into BHIG broth 5% DMSO, with or without test compounds (20 μM JBD1, 20 μM ANG1, 20 μM JBD1 and 100 μM MK-7, or 100 μM MK-7), and 2-mL aliquots were cultured in 12-well flat-bottomed polystyrene plates (Corning) at 37°C for 4 or 24 h under static conditions. Cells from 1 mL of the culture were harvested by centrifugation at 5,000 × *g* and 25°C for 10 min and resuspended in 1 mL of PBS. Then, 1 μL of component A (preliminarily diluted 10-fold with DMSO) included in the BacLight RedoxSensor Green Vitality Kit (Invitrogen) was added to the cells and incubated for 10 min at room temperature. Cells were collected by centrifugation, fixed in 4% PFA for 5 min at room temperature, washed twice, and resuspended in 1 mL of PBS. The cell suspensions were diluted 10-fold with DDW, and stained cells were analyzed using a flow cytometer (CytoFLEX, Beckman Coulter, CA, USA) at an excitation wavelength of 488 nm and an emission wavelength of 525 nm. Cells were flowed at 10 μL/min, and 10,000 events were recorded for each sample.

### PIA dot blot.

S. aureus SH1000 cells cultured in BHIG broth containing 5% DMSO for 24 h in the presence or absence of the test compound (20 μM JBD1, 20 μM JBD1 and 100 μM MK-7, 20 μM JBD1 and 100 μM UQ, or 100 μM MK-7). After harvesting cells by centrifugation, ECMs were extracted based on previously described methods (50). Dot blot analysis of PIA was performed according to a previously published method (28). ECM samples corresponding to the amount extracted from 100 μg of cells were used.

### Transcriptome analysis.

S. aureus SH1000 was grown under biofilm conditions for 4 h in the presence or absence of the test compounds (20 μM JBD1 or 20 μM JBD1 and 100 μM MK-7). Cells were harvested by centrifugation at 5,000 × *g* at 25°C for 10 min and immediately incubated with an appropriate volume of RNAprotect Bacteria Reagent (Qiagen, Hilden, Germany) for 5 min. Cells were collected by centrifugation at 5,000 × *g* at 25°C for 10 min and stored at −80°C. Total RNA was extracted using an RNeasy Midi Kit (Qiagen) according to the manufacturer’s instructions, except that cells were treated with 0.2 mg/mL lysostaphin (Wako) at 37°C for 30 min before extraction. The extracted total RNA samples were stored at −80°C until use. GEBEWIZ (https://www.genewiz.com/) performed RNA-seq including a quality check of RNA samples, stranded library preparation with rRNA depletion, sequencing using Illumina HiSeq (2 × 150 bp), and data analysis. Data were aligned to S. aureus NCTC 8325 reference genome (NC_007795.1) via software Bowtie2 (51). The accession number for the RNA-sequencing data set reported in this paper is DRA012491. Primers for real-time PCR listed in Table S4 were designed according to the published sequences of S. aureus genes, using Primer Express 3.0.1 software (Applied Biosystems, Foster City, CA, USA). Real-time PCR was performed using a Step One Plus (Applied Biosystems) and Power SYBR green PCR master mix (Applied Biosystems). Reactions (20 μL per sample) were performed in triplicate using 96-well plates. All reactions contained 5 ng of cDNA, 10 μL of Power SYBR green Master Mix, 0.2 μM of each primer, and nuclease-free water. According to a previous report, transcript levels of *hu* were used to normalize the transcript levels (52). Amplification was performed with the following program: 10 min at 95°C, 35 cycles for 15 s at 95°C, and 60 s at 60°C. After PCR, melt curve analysis was performed as follows: 15 s at 95°C, 60 s at 60°C, and 15 s at 95°C. Relative gene expression levels were calculated according to the differences in cycle thresholds (Δ*C_T_*) of all samples (53).

### Metabolome analysis.

Metabolites were extracted from S. aureus cells based on the protocol provided by Human Metabolome Technologies (HMT; Yamagata, Japan), with modifications. Briefly, S. aureus SH1000 was cultured in 50 mL of BHIG broth containing 5% DMSO for 8 h in the presence or absence of compounds (100 μM JBD1, 20 μM JBD1, 20 μM JBD1 and 100 μM MK-7, and 100 μM MK-7). Cells were harvested by centrifugation at 5,800 × *g* at 4°C for 5 min. To remove ECM molecules that cause clogging during subsequent filtration (especially PIA), the cells were washed with 10 mL of 1.5 M NaCl as described previously (50). Then, the cells were washed twice with 10 mL of DDW. The number of cells corresponding to the value obtained by multiplying OD_595_ with the sample volume in mL (OD·mL) of 20 was collected and resuspended in 1.6 mL of methanol by sonication. After addition of an internal standard solution (HMT), cell suspensions were centrifuged at 2,300 × *g* at 4°C for 5 min. The supernatants were then filtered through a cellulose membrane filter (UltrafreeMC-PLHCC for metabolome analysis, HMT). The extracted metabolite samples were stored at −80°C until use. Targeted quantitative analysis of metabolites using capillary electrophoresis time of flight mass spectrometer was performed by HMT as described previously (54–56). The 110 major metabolites listed in Table S4 were quantified based on the concentration of the internal standard. PCA and heatmap analysis were performed using SampleStat ver. 3.14 and PeakStat ver. 3.18 software (HMT), respectively.

### Quantification and statistical analysis.

Data were analyzed using GraphPad Prism 7 software (GraphPad, San Diego, CA, USA). Statistical analysis with one-way ANOVA or Student's *t* test was performed. All *P* values <0.05 were considered statistically significant.
