# Supplementary material for: Small-Molecule-Induced Activation of Cellular Respiration Inhibits Biofilm Formation and Triggers Metabolic Remodeling in Staphylococcus aureus
Source: mBio. 2022 Jul 19;13(4):e00845-22. doi: 10.1128/mbio.00845-22 (PMC9426486; doi:10.1128/mbio.00845-22)
Supplement: FIG S1 [file mbio.00845-22-s0001.pdf]

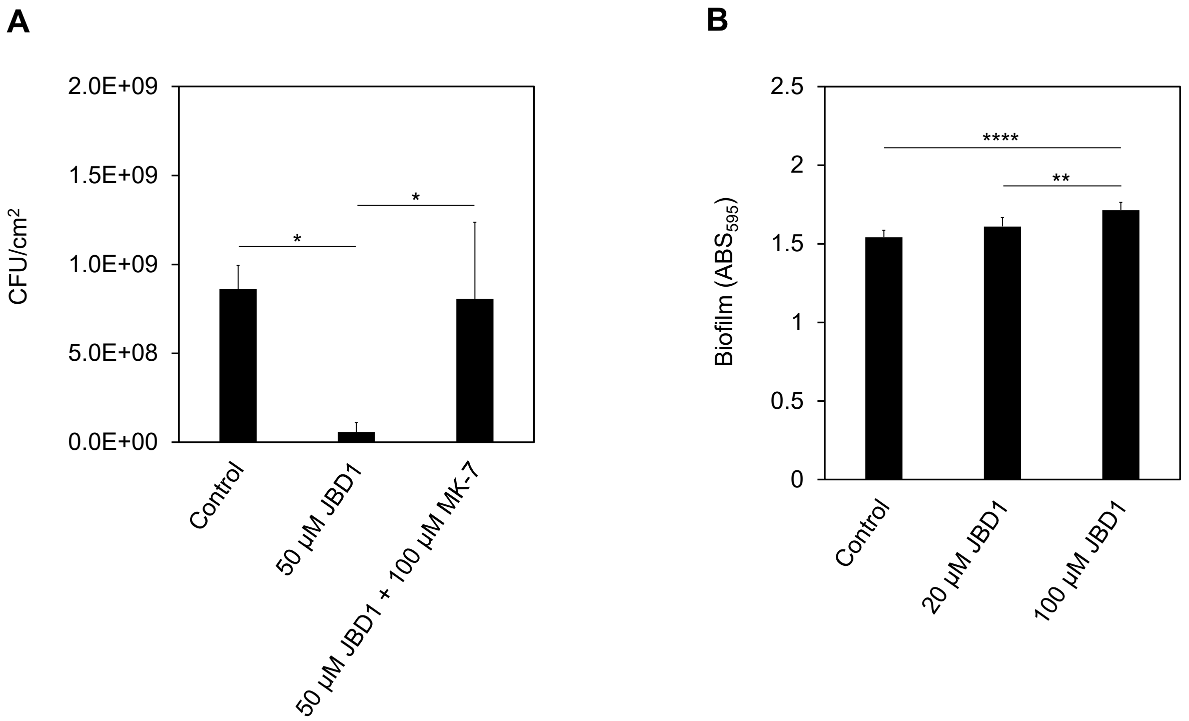

Figure S1. Effects of JBD1 on colony-forming units (CFUs) of biofilms (A) and stability of mature biofilms (B)

(A) Biofilms of *S. aureus* SH1000 were formed in the presence and absence of JBD1 and MK-7, and the CFUs were measured. Mean values were compared via one-way ANOVA. *$p < 0.05$ (Tukey's multiple comparison test).

(B) Mature biofilms of *S. aureus* SH1000 were treated with JBD1. The quantitative values of the crystal violet-stained biofilms on 96-well plates are shown. Data represent the means with standard error from three independent experiments. Mean values were compared via one-way ANOVA. **$p < 0.01$, ****$p < 0.0001$ (Tukey's multiple comparison test).
