# Supplementary material for: Small-Molecule-Induced Activation of Cellular Respiration Inhibits Biofilm Formation and Triggers Metabolic Remodeling in Staphylococcus aureus
Source: mBio. 2022 Jul 19;13(4):e00845-22. doi: 10.1128/mbio.00845-22 (PMC9426486; doi:10.1128/mbio.00845-22)
Supplement: FIG S2 [file mbio.00845-22-s0003.pdf]

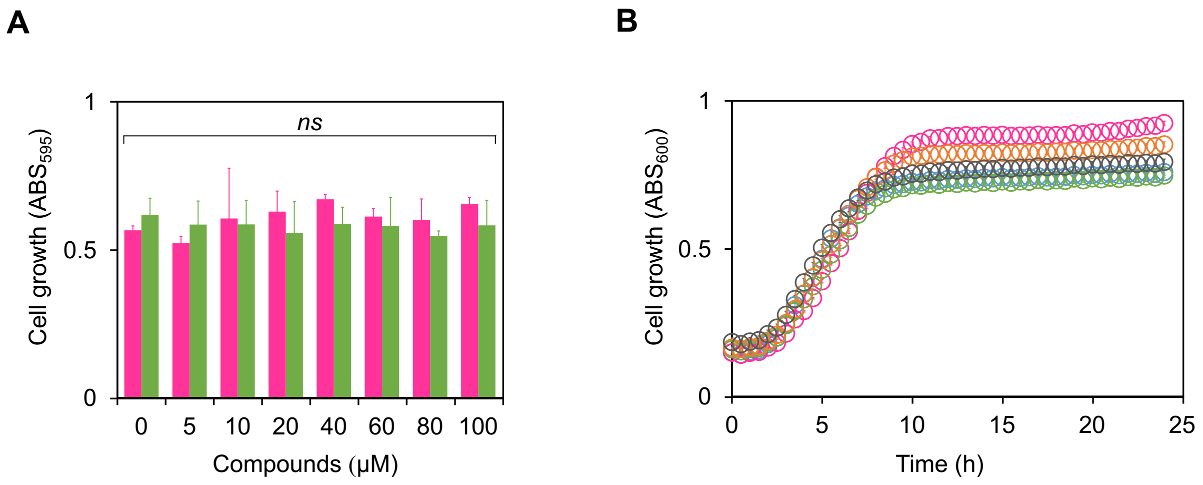

Figure S2. Effects of JBD1 and ANG1 on cell growth

(A) Absorbance of *S. aureus* SH1000 after culturing for 24 h in the presence or absence of JBD1 (magenta) or ANG1 (green). Data represent the means of three independent experiments with standard error. Mean values were compared against 0 µM via one-way ANOVA.

(B) Growth curves of *S. aureus* SH1000 in the presence of 5% DMSO (blue), 20 µM JBD1 (magenta), 20 µM ANG1 (green), 20 µM JBD1 and 100 µM MK-7 (orange), and 100 µM MK-7 (gray). Data represent the means of three independent experiments with standard error (error bars are smaller than the size of the symbols).
