# Supplementary material for: Small-Molecule-Induced Activation of Cellular Respiration Inhibits Biofilm Formation and Triggers Metabolic Remodeling in Staphylococcus aureus
Source: mBio. 2022 Jul 19;13(4):e00845-22. doi: 10.1128/mbio.00845-22 (PMC9426486; doi:10.1128/mbio.00845-22)
Supplement: FIG S3 [file mbio.00845-22-s0005.pdf]

JBD1 (15.4)

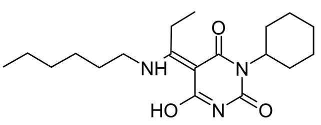

ANG1 (>100)

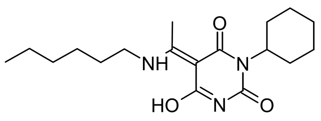

ANG2 (54.8)

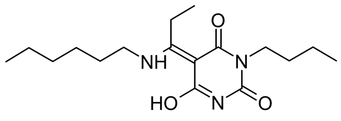

ANG3 (>100)

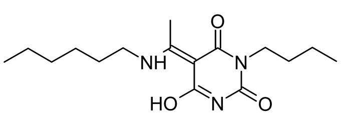

ANG4 (>100)

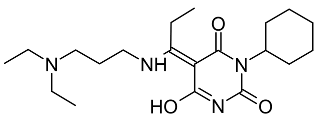

ANG5 (>100)

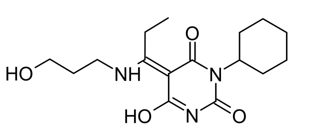

ANG6 (>100)

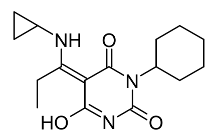

ANG7 (>100)

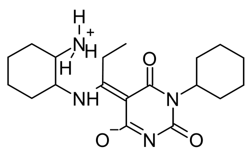

ANG8 (>100)

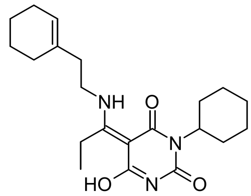

ANG9 (>100)

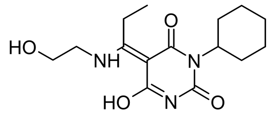

ANG10 (>100)

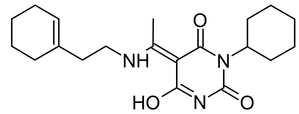

ANG11 (>100)

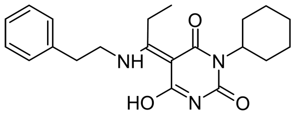

ANG12 (>100)

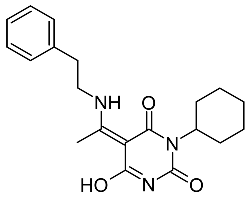

ANG13 (>100)

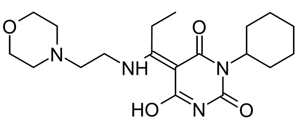

ANG14 (>100)

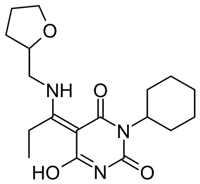

ANG15 (>100)

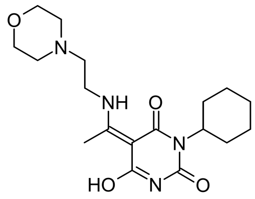

ANG16 (>100)

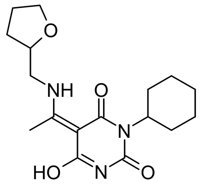

ANG17 (>100)

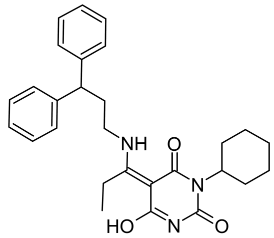

ANG18 (>100)

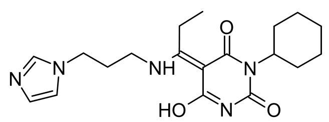

ANG19 (>100)

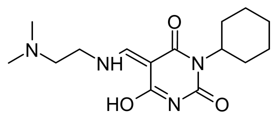

ANG20 (39.0)

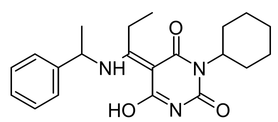

ANG21 (>100)

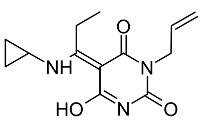

ANG22 (>100)

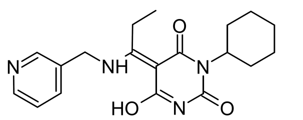

ANG23 (>100)

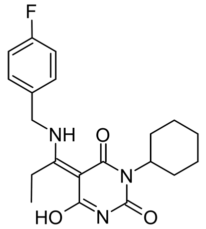

ANG24 (>100)

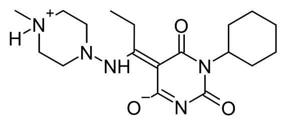

Figure S3. Structure-activity relationships of biofilm inhibitors

Chemical structures of JBD1 and 24 structural analogs . The values in parentheses indicate biofilm-inhibitory activity against *S. aureus* SH1000 (IC_{50}, μM).
