# Supplementary material for: Small-Molecule-Induced Activation of Cellular Respiration Inhibits Biofilm Formation and Triggers Metabolic Remodeling in Staphylococcus aureus
Source: mBio. 2022 Jul 19;13(4):e00845-22. doi: 10.1128/mbio.00845-22 (PMC9426486; doi:10.1128/mbio.00845-22)
Supplement: FIG S4 [file mbio.00845-22-s0007.pdf]

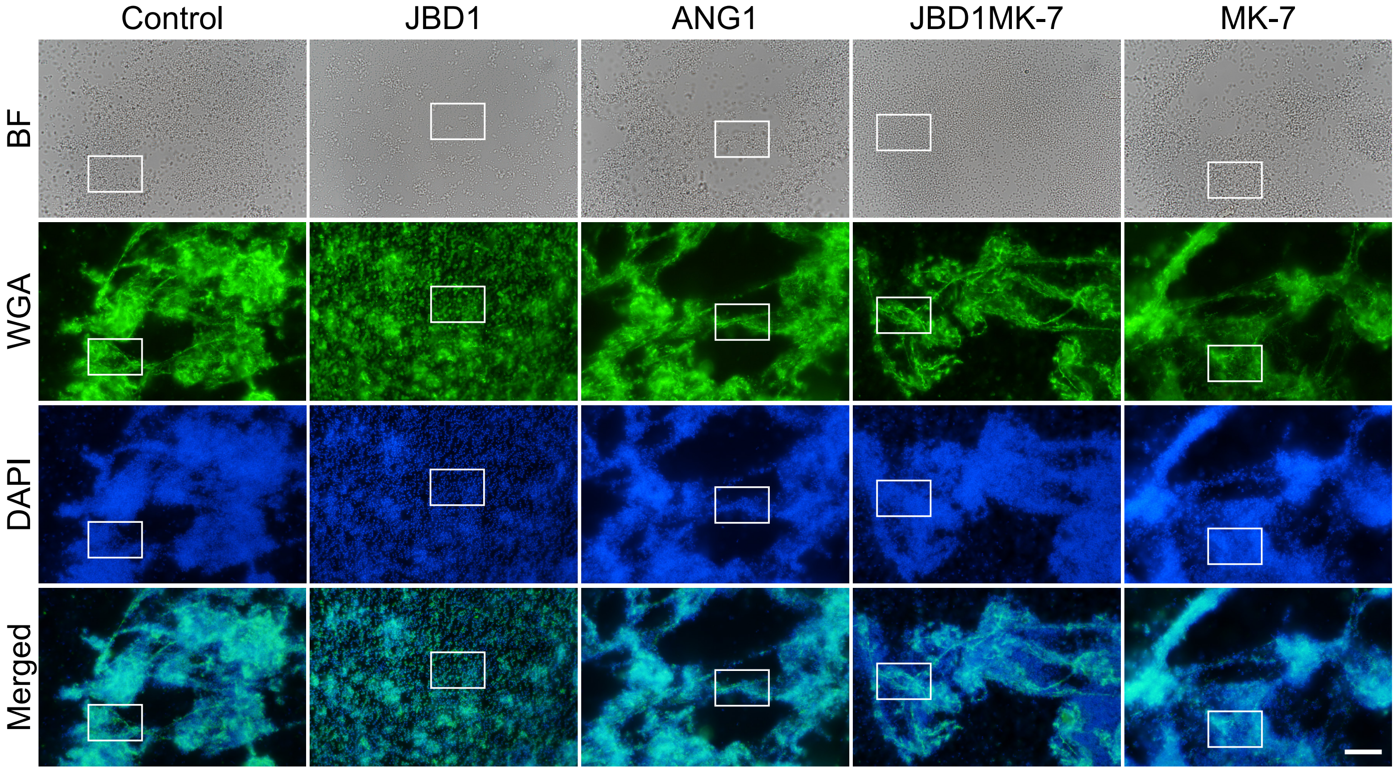

Figure S4. Imaging of fluorescently labeled PIA
PIA and DNA of *S. aureus* SH1000 were stained with WGA-Alexa488 and DAPI, respectively. Each panel in Fig. 1C is an enlargement of the white frame. Bar, 20 µm.
