# Supplementary material for: Small-Molecule-Induced Activation of Cellular Respiration Inhibits Biofilm Formation and Triggers Metabolic Remodeling in Staphylococcus aureus
Source: mBio. 2022 Jul 19;13(4):e00845-22. doi: 10.1128/mbio.00845-22 (PMC9426486; doi:10.1128/mbio.00845-22)
Supplement: FIG S5 [file mbio.00845-22-s0009.pdf]

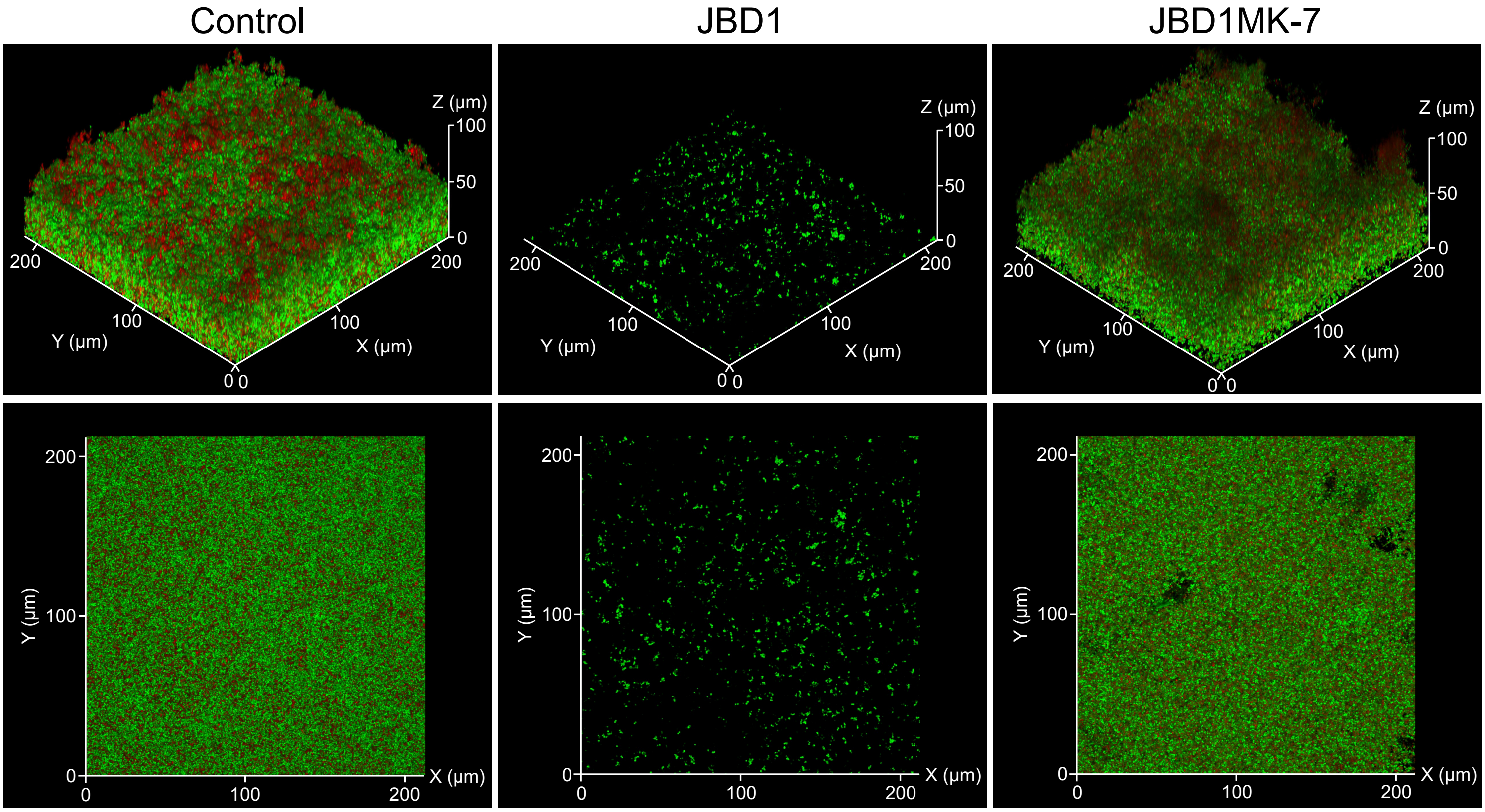

Figure S5. Confocal laser scanning microscopy (CLSM) images of biofilms
Biofilms of *S. aureus* SH1000 formed in the absence of a compound (the control) and the presence of 50 µM JBD1 (JBD1) or 50 µM JBD1 and 100 µM MK-7 (JBD1MK-7) were stained with SYTO9 (green) and PI (red), which stain live and dead cells, respectively. Three-dimensional structures of the biofilms were observed using CLSM.
