# Supplementary material for: Small-Molecule-Induced Activation of Cellular Respiration Inhibits Biofilm Formation and Triggers Metabolic Remodeling in Staphylococcus aureus
Source: mBio. 2022 Jul 19;13(4):e00845-22. doi: 10.1128/mbio.00845-22 (PMC9426486; doi:10.1128/mbio.00845-22)
Supplement: TABLE S1 [file mbio.00845-22-s0002.pdf]

Table S1. MICs (µg/ml) of antibiotics against *S. aureus* strains in the presence or absence of compounds.

| Strain | Antibiotic | Control | JBD1 | ANG1 | JBD1MK-7 | MK-7 |
|---|---|---|---|---|---|---|
| MS9 | Gentamicin | 8 | 2 | 8 | 4 | 16 |
| | Kanamycin | 64 | 8 | 64 | 32 | 64 |
| | Neomycin | 64 | 8 | 64 | 32 | 64 |
| | Tobramycin | 16 | 2 | 16 | 8 | 16 |
| MR4 | Gentamicin | 16 | 2 | 8 | 8 | 16 |
| | Kanamycin | 64 | 8 | 64 | 32 | 64 |
| | Neomycin | 64 | 8 | 64 | 32 | 64 |
| | Tobramycin | 16 | 2 | 16 | 8 | 16 |
| MR10 | Gentamicin | 1024 | 256 | 512 | 512 | 512 |
| | Kanamycin | >2048 | 2048 | >2048 | >2048 | >2048 |
| | Neomycin | >2048 | 1024 | >2048 | >2048 | >2048 |
| | Tobramycin | >2048 | >2048 | >2048 | >2048 | >2048 |
| USA300 | Gentamicin | 32 | 4 | 16 | 32 | 32 |
| | Kanamycin | >2048 | >2048 | >2048 | >2048 | >2048 |
| | Neomycin | >2048 | >2048 | >2048 | >2048 | >2048 |
| | Tobramycin | 32 | 8 | 32 | 16 | 32 |
