# Supplementary material for: Small-Molecule-Induced Activation of Cellular Respiration Inhibits Biofilm Formation and Triggers Metabolic Remodeling in Staphylococcus aureus
Source: mBio. 2022 Jul 19;13(4):e00845-22. doi: 10.1128/mbio.00845-22 (PMC9426486; doi:10.1128/mbio.00845-22)
Supplement: FIG S6 [file mbio.00845-22-s0010.pdf]

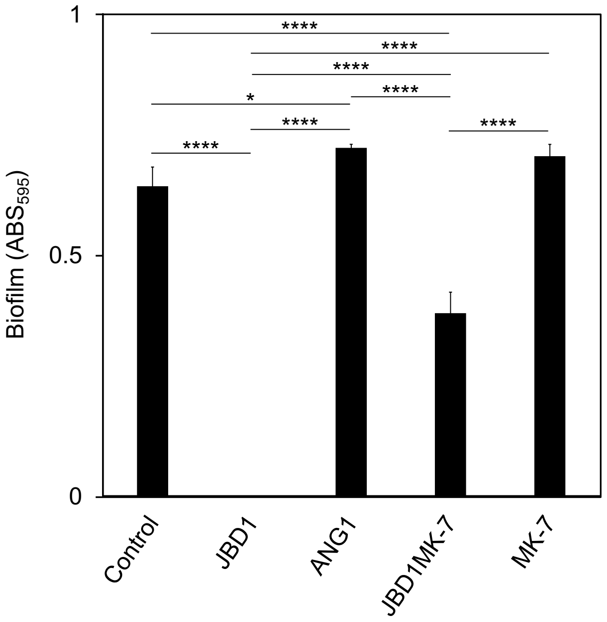

Figure S6. Effects of compounds on biofilm formation at 4 hours of growth
S. aureus SH1000 biofilms were formed for 4 hours in the presence of 5% DMSO (the control), 20 µM JBD1 (JBD1), 20 µM ANG1 (ANG1), 20 µM JBD1 and 100 µM MK-7 (JBD1MK-7), and 100 µM MK-7 (MK-7). The quantitative values of the crystal violet-stained *S. aureus* SH1000 biofilms formed on 96-well plates are shown. Mean values were compared via one-way ANOVA. *$p < 0.05$, ****$p < 0.0001$ (Tukey's multiple comparison test).
